# SENIOR MODELS: SUCCESSFUL AGING AND SERIOUS LEISURE

**DOI:** 10.1093/geroni/igz038.2651

**Published:** 2019-11-08

**Authors:** Sanghee Lee, Jaeyoon Bae, Sua Im, Jinmoo Heo

**Affiliations:** Yonsei University, Seoul, Korea, Republic of

## Abstract

Serious leisure involves productive engagement and commitment in leisure activities. Literature shows that participating in serious leisure is associated with physical and mental health benefits of older adults. The behavior of senior modeling reflects serious leisure engagement that might offer a new insight useful in understanding successful aging. We explored the experience of senior models as a form of serious leisure. Using selective optimization with compensation as well as serious leisure framework, we attempted to identify how senior modeling activity contributes to successful aging. This study used in-depth interviews using purposeful sampling, and data were collected over two months in 2019. The participants were 31 senior models (average age = 67). The analysis resulted in three themes which contained characteristics of serious leisure as well as selective optimization with compensation: identifying new possibilities, serious engagement, and rewards from meaningful experiences. This study demonstrated various experiential characteristics associated with modeling as a form of serious leisure. Through selection, optimization, and compensation process, the participants seemed to achieve successful aging. We found that senior model experiences promoted active lifestyle, health benefits, and interpersonal relationships. To our knowledge, this is the first exploration of the experience of senior modeling activity. Consistent with existing literature, our study provides evidence of the significant role of serious leisure in later life. We suggest that senior modeling program holds promise as an effective way for older adults because it can be used as a self-care approach and community programs not only in Korea, but at various locations.

